# Evaluation of the Effect of Parenting Style and Parental Mealtime Actions on the Eating Behavior of Children with Epilepsy

**DOI:** 10.3390/nu16091384

**Published:** 2024-05-02

**Authors:** Tutku Balcı, Nihan Çakır Biçer, Hande Gazeteci Tekin, Pınar Edem

**Affiliations:** 1 Department of Nutrition and Dietetics, Institute of Health Sciences, Acibadem Mehmet Ali Aydinlar University, 34752 Atasehir, Istanbul, Turkey; tutkumbalci@gmail.com; 2 Department of Nutrition and Dietetics, Faculty of Health Sciences, Acibadem Mehmet Ali Aydinlar University, Içerenköy Mahallesi Kayisdagi Caddesi No. 32, 34752 Atasehir, Istanbul, Turkey; 3 Division of Pediatric Neurology, Department of Pediatrics, Faculty of Medicine, Izmir Bakircay University, 35665 Menemen, Izmir, Turkey; gazetecihande@yahoo.com.tr (H.G.T.); pinaredem@gmail.com (P.E.)

**Keywords:** children, parents, childhood epilepsy, parenting style, parental eating-time actions, parents’ mealtime behaviors, eating behavior, diet quality, growth, parenting practices

## Abstract

Background: Research on the interaction of parenting style, parents’ mealtime behaviors, and children’s eating behavior in the presence of chronic disease is limited. This study aimed to investigate the impact of parenting style and parental mealtime actions on the eating behavior of children with epilepsy. Methods: Thirty-one children with epilepsy, thirty-one healthy children (aged 4–9 years), and their parents were included. The Multidimensional Assessment of Parenting Scale (MAPS), Parent Mealtime Action Scale, Children’s Eating Behavior Questionnaire, and Healthy Eating Index (HEI)-2015 were applied. The MAPS, HEI-2015 scores, and body mass index for age Z scores were similar in both groups (*p* > 0.05). In the epilepsy group, the food approach behavior score was higher, and positive correlations were noted between broadband negative parenting and food approach behavior, and the HEI-2015 score and broadband positive parenting (*p* < 0.05). Regression analysis showed that broadband negative parenting and snack modeling increased the food approach behavior in the epilepsy group. Owing to the chronic disease, the effects of parent–child interaction on the child’s eating behavior in the epilepsy group differed from those of healthy children reported in the literature.

## 1. Introduction

One of the most prevalent chronic neurological diseases is childhood epilepsy. Epilepsy is characterized by a persistent predisposition to seizures and abnormal brain activity that causes unusual behavior, sensations, and, sometimes, loss of awareness, with neurobiological, cognitive, psychological, and social consequences. Epilepsy affects approximately 50 million individuals worldwide, accounting for a significant proportion of the disease burden [1]. The incidence of epilepsy ranges from 41 to 187/100,000. A higher incidence is reported in less developed countries, especially in rural areas [2]. The prevalence of epilepsy is 0.4–0.5% in Europe and North America. In Turkey, this rate varies between 0.5% and 1.2%; however, in a study conducted across Turkey, the prevalence was reported as 0.8%, which is higher than that in developed countries [3,4,5,6]. The cause of epilepsy includes genetic, structural, metabolic, infective, and neuroimmune causes. Knowledge of the etiology is essential for treatment, follow-up, and the prediction of morbidity. To diagnose epilepsy, a wide range of clinical and laboratory tests are used. Along with detailed anamnesis, electroencephalography (EEG), and brain-imaging methods to determine the seizure semiology of patients with epilepsy, metabolic and genetic tests have gained significance in increasing the diagnostic probability. Anti-seizure treatments of patients who are followed up with a diagnosis of epilepsy are adjusted according to the epilepsy type, EEG findings, underlying etiology, and prescribed duration of drug use [7]. Although seizure control can be achieved in two-thirds of patients without significant side effects with the appropriate use of medication, pharmacologic treatments may not frequently be sufficient. Epilepsy resistant to medical treatment is considered in most cases wherein seizure cessation is not achieved with two to three properly selected and appropriately dosed antiepileptic drugs. In this case, the treatment options include epilepsy surgery, electrical vagus nerve stimulation, or ketogenic diet therapy [8]. Although the mechanism of action of the ketogenic diet is not clearly known, it is also known that recurrent seizures induce oxidative stress and neuroinflammatory changes. The ketogenic diet is thought to improve the stress response and inflammation by suppressing seizures and associated neuronal damage through its metabolites [9]. In the ketogenic diet, most of the daily energy (>50%) is provided by fat and there are four different types of ketogenic diets: the classic long-chain triglyceride (LCT) ketogenic diet, medium-chain triglyceride (MCT) ketogenic diet, modified Atkins diet (MAD), and low glycemic index treatment [10]. Short-term complications of the ketogenic diet include gastrointestinal problems including constipation, vomiting, diarrhea, hunger, abdominal pain, gastroesophageal reflux, and fatty diarrhea; long-term consequences include impaired kidney function, decreased bone mineral density, and increased blood lipid levels [11]. In addition, nutritional deficiencies in fiber, vitamin D, B group vitamins, calcium, and trace elements may be observed [12].

Epileptic seizures and epilepsy treatment negatively affect the patient and family/caregivers in terms of quality of life and various social aspects [13,14,15,16,17]. Parents of chronically ill children take on more child-rearing obligations than parents of healthy children, particularly responsibilities such as meeting the child’s physical treatment needs, may experience financial difficulties, and may experience feelings of inadequacy, anger, anxiety, worry, and depressive symptoms [18,19,20]. Parents of children with epilepsy have fears regarding the unpredictability of epileptic symptoms, concerns regarding their children’s physical and mental health, losing their children, being exposed to sibling or peer bullying owing to their differences, having learning difficulties, and not gaining independence in the future [21]. Epilepsy is, therefore, not only a medical condition but also a social problem for children and their parents [22].

Parents may perceive epilepsy as a danger to their children and may have an urge to protect their children from the disease and seizures. This impulse can cause parents to show positive or negative reactions; shape their approach to their children, their behaviors, and attitudes; and affect their parenting practices [23]. Even in the management of simple daily activities including sleeping behaviors, school life, and sports activities, differences and problems are observed. Consequently, although the experiences and concerns of parents during the medical management of epilepsy and seizures affect their behavior and parenting style, the family’s approach to the child can also affect the child’s daily life and behavior [21]. Nutrition is a field wherein parental behavior can also shape the child’s behavior. Although parents can significantly impact their children’s nutrition and, ultimately, body weight, the relationships underlying this effect have not yet been clearly defined [24].

Parenting style is a general behavioral structure that determines the emotional context wherein parents and children interact and reflects the emotional climate wherein children are raised [25,26,27]. This style comprises the following two dimensions: demandingness/control and responsiveness/nurturance. With the intersection of these two dimensions, four parenting styles emerge: (1) the authoritative parenting style includes high demandingness and high responsiveness behaviors and is associated with parental involvement, nurturance, and expectations with monitoring; (2) the authoritarian parenting style involves high demandingness and low responsiveness behaviors and is accompanied by restrictive, punitive, and power-assertive behaviors; (3) the indulgent parenting style includes low demandingness and high responsiveness behaviors, and an attitude of warmth and acceptance toward the child’s behavior is dominant; (4) the uninvolved parenting style is characterized by low demandingness and low responsiveness behaviors and is associated with little control, nurturance, or involvement with the child [28,29,30]. Inconsistent results have been reported in the literature regarding the relationship between parenting style and children’s eating behavior and weight status [31,32,33,34]. Several studies have reported that children of parents with an authoritative parenting style are associated with consuming healthy foods, including dairy products, fruits, and vegetables, and having a healthier body mass index (BMI) [31,34,35].

Parenting practices are “behaviors defined by specific contexts and socialization goals” [36]. Food parenting practices are parenting practices related to the child’s nutrition and eating behaviors [31]. The child’s eating behavior is influenced by parenting practices. Vaughn stated that there are three basic food parenting practices: structure, coercive control, and autonomy support [37]. The structure is associated with rules and boundaries and refers to meal and snack routines, food availability, and accessibility. The coercive control refers to practices associated with negative relationships, including restriction, forcing to eat, threats, and rewards/bribes. Conversely, the autonomy support includes practices that include encouragement and nutrition-related education and is associated with more healthy and less unhealthy food consumption [37]. Offering food variety at home, regularly exposing the child to new and familiar foods, verbal encouragement, and parental modeling of eating are successful parenting strategies for enhancing food acceptability in children. However, parental mealtime practices such as controlling food intake, suppressing consumption, restricting foods, and employing rewards negatively impact children’s eating behavior. These techniques can result in lower food acceptance and aversion to items that children are pressured to eat [25]. Although there are studies in the literature, mostly cross-sectional, regarding the relationship between food parenting practices such as restricting the type and amount of food and using food as a reward and the child’s eating behavior and weight status, fewer studies have evaluated these relationships with parenting styles in a broader context [38]. Conversely, studies examining parent–child relationships in terms of nutritional behavior in children with chronic diseases are limited [18]. In studies conducted among healthy children, in addition to the role of parents in influencing the child’s eating behavior, a two-way interaction between the parent’s mealtime actions and the child’s eating behavior was observed [39,40]. Increased parental stress, problems in maintaining routines, and inappropriate mealtime interactions can occur owing to a child’s negative eating behavior [41]. This complex bidirectional interaction influences the child’s eating behavior, thereby contributing to the mealtime experience of both the child and parent [42].

The acquisition of healthy eating behaviors in children with epilepsy is important in terms of diet quality, and, thus, the maintenance of growth development and the development of cognitive functions. The role of parental behaviors in shaping feeding behaviors should not be ignored. Parenting skills and the parent–child relationship can be influenced by raising a child with a chronic illness [18]. Although the mechanism of the change in eating behaviors in epilepsy is not known, it is thought that the interaction between parent and child affects this mechanism [43]. It has been reported that eating disorder symptoms are more common in adolescent epilepsy patients and their nutritional status is worse than their peers [44]. To the best of our knowledge, there is only one study in the literature evaluating eating behavior in children with epilepsy; however, in this study, the relationship between children’s eating behaviors and any variable that may affect these behaviors was not evaluated [43]. In addition, there are no studies in the literature examining the relationship between parents’ mealtime actions and children’s eating behavior. This study was designed with the foresight that the relationship between food parenting practices and eating behaviors between children with epilepsy and their parents may change, an interaction different from the relationship between healthy children and their parents may arise, and this interaction may affect the child’s diet quality and growth. This study aimed to evaluate the effect of parenting style and parental mealtime actions on the eating behavior of children with epilepsy.

## 2. Materials and Methods

### 2.1. Study Design and Participants

Between October and December 2023, this cross-sectional case–control study was conducted in the Pediatric Neurology Outpatient Clinic and Pediatrics Outpatient Clinic of İzmir Bakırçay University Çiğli Training and Research Hospital with children diagnosed with epilepsy and healthy children aged 4–9 years and their parents (40 mothers, and 22 fathers). The children with epilepsy constituted the epilepsy group (*n* = 31) and the healthy children constituted the control group (*n* = 31). The inclusion criteria for all children and their parents were that the parents did not have a psychiatric or mental illness that would prevent them from understanding the study-specific data collection form and that the parents gave their consent for participation in the study. In addition, the inclusion criteria for the group with epilepsy were that the child was between the ages of 4 and 9 years, had no disease other than epilepsy, and was not on any medical nutrition therapy for seizure control, including classic LCT ketogenic diet, MCT ketogenic diet, MAD, and low glycemic index treatment. The exclusion criteria were that the child with epilepsy was younger than 4 years of age or older than 9 years of age, had another concomitant disease, and was applying any type of ketogenic diet for seizure control. The inclusion criteria for the healthy child group were that the child was between the ages of 4–9 and did not have any chronic disease. The exclusion criteria for healthy children were that the child was younger than 4 years of age or older than 9 years of age and had any chronic disease.

This study was approved by the Acıbadem Mehmet Ali Aydınlar University and Acıbadem Healthcare Institutions Medical Research Ethics Committee (ATADEK) (ATADEK-2023/13, 17 August 2023) and conducted in accordance with the Declaration of Helsinki. All participants provided written informed consent.

### 2.2. Demographic and Medical Data

In the first section of the data collection form prepared for the study, the demographic characteristics of the parent and child including age, sex, educational level, marital status, employment status, income, spousal consanguinity, and health status were recorded. The number of main meals and the picky eating behavior of the children were examined. Moreover, medical information such as age at diagnosis, pharmacologic treatment, and seizure status of the child with epilepsy were recorded.

### 2.3. Anthropometric Measurements

Anthropometric data (body weight and height) of the child and parents were measured and recorded by the researchers according to the World Health Organization (WHO) guidelines, and BMI was calculated. The anthropometric measurements of the children were evaluated using WHO age- and sex-specific growth standards, and Z scores were calculated [45].

### 2.4. Multidimensional Assessment of Parenting Scale (MAPS)

Parent and Forehand (2017) developed the MAPS (Parent Form) to assess parenting styles [46]. Karababa (2019) performed the Turkish language adaptation of the MAPS [47]. Comprising 34 items, the MAPS includes 7 subscales: (1) The proactive parenting subscale is associated with appropriate child-centered responses for preventing anticipated difficulties or potential conflicts. (2) The positive reinforcement subscale of parenting includes indicators of approval, such as praise and congratulations when the child fulfills his/her responsibilities or after any positive behavior. (3) The warmth subscale includes indicators of love, affection, and fondness. (4) The supportiveness subscale measures parental involvement in their child, receptivity to their ideas and perspectives, and attitudes and behaviors that promote constructive communication. (5) The hostility subscale encompasses unfavorable parenting, which is parent-centered and overly controlling, and harshness, which includes compelling practices such as arguing, threatening, ineffective disciplinary, and irritability behaviors. (6) The lax control subscale refers to the parents’ lack of control over their children or their permissive attitude and behavior toward their children. (7) The physical control subscale measures general and specialized physical discipline aside from anger and annoyance. The MAPS can be evaluated in the following two subdimensions: (1) broadband positive parenting with the combination of proactive parenting, positive reinforcement, warmth, and supportiveness subscales; and (2) broadband negative parenting with the combination of hostility, lax control, and physical control subscales. The MAPS items are scored on a five-point Likert scale (1, never; 5, always), and subscale scores are obtained by summing the item scores. The MAPS is administered to parents of children and adolescents aged 4–17 years [47]. In this study, the Cronbach alpha (α) coefficient value of the MAPS was 0.71.

### 2.5. Parent Mealtime Action Scale (PMAS)

Hendy et al. (2009) developed the PMAS to assess the behaviors of parents while feeding their children [48]. Arslan and Erol (2014) performed the Turkish language adaptation of the PMAS [49]. The PMAS can be administered to parents of children between the ages of 4 and 12, consists of 31 items, and is a three-point Likert-type scale. The scale has no total score, and each subscale is evaluated separately. The PMAS comprises nine subscales: (1) The snack limits subscale is associated with the limitations parents place on the amount of food, drinks, and snacks their children eat. (2) The positive persuasion subscale reveals the verbal activities parents employ to encourage their children to eat. (3) The daily fruit–vegetable availability subscale refers to parents’ behaviors regarding how often they consume fruits and vegetables for themselves and their children. (4) The use of rewards subscale assesses the activities or foods offered by parents to entice their children to eat. (5) The insistence on eating subscale shows the pressuring attitudes and behaviors of parents to get their children to eat. (6) The snack modeling subscale shows parents’ behaviors regarding how often they consume snack foods. (7) The special meals subscale refers to whether meals different from the meals eaten by all family members are prepared for the child. (8) The fat reduction subscale measures parents’ behavior in using animal fats. (9) The many food choices subscale captures whether parents allow their children to make choices about their food intake [49]. In this study, the Cronbach alpha (α) coefficient value of the PMAS was 0.60.

### 2.6. Children’s Eating Behavior Questionnaire (CEBQ)

To assess children’s eating behavior, the CEBQ was developed by Wardle et al. (2001) [50]. Yilmaz et al. (2008) performed the Turkish language adaptation of the CEBQ [51]. The questionnaire, comprising 35 items answered by parents, is a five-point Likert scale (1, never; 5, always) and can be administered to parents of children aged 2–9 years. The CEBQ has eight subscales: (1) The food responsiveness subscale describes the child’s interest in food. (2) The emotional overeating subscale associates the child’s emotional state with overeating. (3) The enjoyment of food subscale includes items related to the child’s passion for eating and appetite. (4) The desire to drink subscale specifically measures interest in beverages. (5) The satiety responsiveness subscale refers to the child’s satiety. (6) The slowness in eating subscale refers to the child’s behavior of eating slowly. (7) The emotional undereating subscale refers to the emotional state reducing eating behavior. (8) The fussiness subscale refers to selective eating behaviors [51].

Eating behaviors in children focus on the following two concepts: food avoidance and food approach. Food avoidant behaviors such as picky eating or fussiness comprise rejection of familiar and novel foods, slowness in eating, emotional undereating, and regulation of eating by internal cues, also known as satiety responsiveness. Conversely, food approach behaviors include food-directed acts and desires, such as emotional overeating, a desire to drink, and responses to external cues such as food enjoyment and food reactivity [42]. Based on this information, to obtain more measurable standard scores and conduct further statistical analyses in this study, the “food approach behaviors” subdimension was formed with the mean scores of the items included in the food responsiveness, emotional overeating, enjoyment of food, and desire to drink subscales; and the “food avoidant behaviors” subdimension was formed with the mean scores of the items included in the satiety responsiveness, slowness in eating, emotional undereating, and fussiness subscales. In this study, the Cronbach alpha (α) coefficient value of the CEBQ was 0.73.

### 2.7. Healthy Eating Index (HEI)-2015

To assess the diet quality of children, HEI-2015 scores were calculated. The HEI-2015 is an a priori index developed by the US Department of Agriculture Center for Nutrition Policy and Promotion to measure adherence to a healthy diet [52]. For the HEI-2015 calculation, an experienced dietician queried the 24 h food consumption record. To determine the portion sizes in food consumption, the Photographic Food Catalog was used [53]. The Beslenme Bilgi Sistemi (the Nutrition Information System) software v.9 (Pasifik Elektronik, Stuttgart, Germany) was used to analyze the energy, macronutrient, and micronutrient intakes of children according to food consumption [54]. The HEI-2015 consists of 13-component scores. A higher score indicates greater compliance with the HEI-2015 [55]. The first 9 of the 12 components establish the diet’s sufficiency, whereas the final 4 determine what should be reduced. The total HEI-2015 score is 100 points, calculated by adding the competence and limited consumption components. The component values range from 0 to 5, 0 to 10, and 0 to 20, with a total score of 100% indicating that the recommended amounts were met or exceeded [56].

### 2.8. Statistical Analysis

Children followed up in the clinics where the study will be conducted constituted the study population. To determine the sample size, a pilot study was conducted with children with epilepsy, healthy children, and their parents who met the inclusion criteria. To calculate the effect size for sample size determination, the calculation (d-value) method developed by Cohen was used [57]. To determine the effect size index d, a pilot study was conducted with 20 participants (10 in each research group). Considering the pilot study data, a difference of approximately 1.2 (±1.7) units was noted in the level of insistence on feeding between parents of children with epilepsy and healthy children. Based on this finding, the effect size level to be used in this study was d = 0.785. In this context, for the quantitative difference between the two groups, d = 0.785, 95% confidence level (1-α), and 80% test power (1-β) using the G-power (version 3.1) package program, the sample group was calculated to be 56 participants (28 in each group). Considering possible losses (approximately 10%), the study was conducted with 62 participants.

All analyses were performed using the Statistical Package for the Social Sciences version 26.0 software (IBM Corp., Armonk, NY, USA). In this study, the Kolmogorov–Smirnov test was used to evaluate the normality assumption of the continuous variables. Categorical and continuous variables were presented as frequency (*n*, %) and as means and standard deviations, respectively. Cronbach alpha (α) coefficient values were determined to measure the reliability of the scales in this study. Comparisons between the two groups in continuous variables were made using the independent-samples *t*-test. The level of relationship between two continuous variables was analyzed using the Pearson correlation test. To determine the effect of independent variables on dependent variables (level of food approach behaviors), multivariate linear regression analysis was employed. A *p*-value of <0.05 was considered statistically significant.

## 3. Results

### 3.1. Demographic Characteristics of the Parents

This study included 31 children with epilepsy, 31 healthy children, and their parents (*n* = 62). The mothers of 19 children with epilepsy (61.3%) and 21 healthy children (67.7%) participated in this study. The mean age and BMI of the parents of the epilepsy and control groups were similar (*p* > 0.05). Here, 51.6% (*n* = 16) and 29.0% (*n* = 9) of the parents in the control and epilepsy groups, respectively, were university graduates, and the educational level of the control group was higher (*p* = 0.024). The distributions of both groups in terms of marital status, employment status, spousal consanguinity, income status, and presence of a diagnosed disease were similar (*p* > 0.05). The demographic characteristics of the parents are presented in Table 1.

### 3.2. Demographic Characteristics of the Children

The demographic characteristics of all children and the medical characteristics of the epilepsy group are shown in Table 2. Here, 51.6% of the epilepsy group (*n* = 16) and 45.1% of the healthy children (*n* = 14) were women. No statistical difference was observed between the mean age and BMI for age Z scores of children with epilepsy and healthy children (*p* > 0.05). The distribution of the number of main meals and the picky eating status did not differ between the groups (*p* > 0.05). The age at diagnosis of children with epilepsy was 62.29 ± 32.98 months. Twenty-three patients with epilepsy (74.2%) used a single medication, and nineteen (61.3%) were primarily cared for by their mothers.

### 3.3. Evaluation of the Scales and HEI-2015 Scores

The mean scales and HEI-2015 scores of the epilepsy and control groups are presented in Table 3. The mean MAPS broadband positive parenting and broadband negative parenting scores of the parents of children with epilepsy were 93.55 ± 8.12 and 32.90 ± 7.38, respectively; the mean scores of the parents of healthy children were 96.42 ± 6.36 and 35.55 ± 9.97, respectively. The parents of children with epilepsy had a significantly higher mean score of the PMAS snack modeling subscale (*p* = 0.044) and a lower mean score of the fat reduction subscale than the parents of healthy children (*p* = 0.020). No statistically significant difference in the mean scores of the groups in the other subscales of the PMAS was noted (*p* > 0.05). The epilepsy group had a higher food approach behavior subdimension score (10.31 ± 2.38) than the control group (9.12 ± 1.98) (*p* = 0.035). Regarding HEI-2015 scores, no significant difference was observed between the two groups (*p* > 0.05).

The difference between the mean scores of the scales and HEI-2015 scores for children with epilepsy according to age (median value below or above 5.4 years), number of antiepileptic drugs used, seizure and food selection status, parents’ age (median value below or above 40 years), sex, and educational status were evaluated (Supplementary Data). In children taking a single antiepileptic drug, the supportiveness subscale score of the MAPS was lower, the slowness in eating subscale score of the CEBQ was higher, and the enjoyment of food subscale score of the CEBQ was higher in children with seizures than in those taking multiple drugs (*p* < 0.05). Children with epilepsy who were picky eaters had higher scores on the warmth subscale of the MAPS, higher scores on the satiety responsiveness and fussiness subscales, and lower scores on the enjoyment of food subscale of the CEBQ (*p* < 0.05) (Table S1, Supplementary Data). Children with epilepsy whose parents’ ages were below 40 years had higher scores in the snack modeling subscale of the PMAS than those whose parents’ ages were above 40 years (*p* < 0.05). Children with epilepsy whose fathers were interviewed had higher scores on the food responsiveness subscale of the CEBQ (*p* < 0.05). Children whose parents had only a primary education had lower scores on the warmth subscale of the MAPS; lower scores on the satiety responsiveness, slowness in eating, and fussiness subscales of the CEBQ; and higher scores on the enjoyment of food subscale of the CEBQ (*p* < 0.05) (Table S2, Supplementary Data).

### 3.4. Evaluation of the Relationship between MAPS, PMAS, and CEBQ Scores

The relationship between the broadband positive parenting and broadband negative parenting subdimensions scores of the MAPS, other subscales, and HEI-2015 scores according to the research groups is shown in Table 4. In the epilepsy group, no correlation was observed between the broadband positive parenting and broadband negative parenting subdimension scores of the MAPS and the subscale scores of the PMAS (*p* > 0.05), whereas, in the control group, a negative correlation was observed between the broadband negative parenting subdimension score and the daily fruit–vegetable availability subscale score of the PMAS (r = −0.404; *p* = 0.024), and a positive correlation was observed between the insistence on eating (r = 0.455; *p* = 0.010) and snack modeling (r = 0.435; *p* = 0.014) subscale scores of the PMAS. In the epilepsy group, a statistically significant positive correlation was noted between the broadband positive parenting subdimension score of the MAPS and the satiety responsiveness subscale of the CEBQ (r = 0.431; *p* = 0.015), and statistically significant positive correlations were observed between the broadband negative parenting subdimension score of the MAPS and the food responsiveness (r = 0.472; *p* = 0.007), emotional overeating (r = 0.449; *p* = 0.011), and food approach behavior subdimensions of the CEBQ (r = 0.450; *p* = 0.011). In the control group, a statistically significant positive relationship was noted between the broadband positive parenting subdimension of the MAPS and the emotional undereating subscale score of the CEBQ (r = 0.372; *p* = 0.040). In the control group, positive and statistically significant relationships were observed between the broadband negative parenting subdimension score of the MAPS and emotional overeating (r = 0.367; *p* = 0.042) and emotional undereating (r = 0.445; *p* = 0.012) subscale and the food avoidant behavior subdimension (r = 0.369; *p* = 0.041) scores of the CEBQ, and the broadband negative parenting subdimension score of the MAPS and the enjoyment of food subscale score of the CEBQ (r = −0.436; *p* = 0.014) showed a negative and statistically significant relationship.

### 3.5. Evaluation of the Relationships between HEI-2015 Scores, BMI for Age Z Scores, and Scale Scores

In the epilepsy group, positive relationships were noted between the HEI-2015 score and the broadband positive parenting subdimension score (r = 0.453; *p* = 0.011) and the positive reinforcement (r = 0.360; *p* = 0.047) and supportiveness subscales (r = 0.376; *p* = 0.037) scores of the MAPS. In the control group, positive correlations were observed between the HEI-2015 score and the snack limits (r = 0.361; *p* = 0.046) and daily fruit–vegetable availability (r = 0.427; *p* = 0.017) subscale scores of the PMAS.

In children with epilepsy, BMI Z scores statistically significantly increased as the CEBQ subscale scores of food responsiveness (r = 0.431; *p* = 0.016) and enjoyment of food (r = 0.470; *p* = 0.008) increased. The relationships between HEI-2015 scores, BMI for age Z scores, and subscale scores according to the research groups are presented in Table 5.

### 3.6. Evaluation of Variables Associated with the Food Approach Behavior of the CEBQ

In the multivariate linear regression analysis conducted to determine the independent factors affecting the level of the food approach behavior score of the CEBQ in children with epilepsy, the variables that had a significant relationship with the level of the food approach behavior score of the CEBQ at a 5% statistical significance level were modeled using the enter method (Table 6). The model was significant at the 5% level (F(10–20) = 2.55; *p* = 0.036). Multicollinearity (variance inflation factor < 5; tolerance > 0.20) and autocorrelation (Durbin–Watson statistic = 2.11) were not detected in the model. In the model, independent variables explain approximately 34% of the total change in the dependent variable (adjusted R^2^ = 0.341). When the relationship between the independent variables of the model and the dependent variable was examined, the broadband negative parenting subdimension score of the MAPS and the snack modeling subscale of the PMAS increased the food approach behavior subdimension score of the CEBQ in children with epilepsy.

## 4. Discussion

Owing to concerns about their children’s health, parents of children with chronic diseases may change their approach to their children and their parenting styles. Epilepsy, one of the most common neurological diseases in childhood, is a significant burden on the child and the family. This study aimed to evaluate the relationship between the parenting style exhibited by parents of children with epilepsy, parental mealtime actions, the child’s eating behavior, diet quality, and growth. In this context, 31 children diagnosed with epilepsy, 31 healthy children, and their parents were evaluated, and some relationships indicated that parents’ positive or negative attitudes play a role in children’s feeding behaviors. Although there are few studies evaluating feeding behaviors in children with chronic diseases, to the best of our knowledge, no studies on the relationship between parental practices and child eating behavior in children with epilepsy have been conducted.

The present study was conducted in a single center, and no significant difference was observed between the distribution of sex, marital status, employment status, income level, and the presence of spousal consanguinity of the children in the epilepsy and control groups (*p* > 0.05). In addition, no significant difference was observed between the mean ages of parents and children (*p* > 0.05). The parents of the epilepsy group had a lower educational level than those of the control group (*p* < 0.05). Similarly, in a study conducted with parents of children with epilepsy and healthy children, no difference was observed between the two groups regarding the mean age of the parents, whereas the parents of the healthy group had a higher educational level than those of children with epilepsy [4]. The fact that this study was conducted in a single center and that the demographic characteristics of the parents were similar enabled the evaluation of a homogeneous group with similar sociocultural characteristics. Considering that sociocultural characteristics may influence parenting style and eating behaviors, this homogeneity may help reduce confounding factors for assessing behavioral traits.

The child’s eating behavior is influenced by several factors and will affect the child’s relationship with food and body weight. Parents, other family members, the family’s lifestyle, and eating attitudes are the main factors affecting a child’s eating behavior. Parents provide food, food environments, and experiences for their children. Children use their parents’ eating behaviors, lifestyle, attitudes toward eating, and satisfaction or dissatisfaction with body image as models for themselves [58].

Parenting style includes various attitudes, beliefs, and behaviors that parents display during their interactions with their children. Positive parenting styles are characterized by warm and close relationships, including positive involvement, whereas negative parenting styles are characterized by strictness, including authoritarian parenting, punitive parenting, overprotective parenting, and rejecting or coddling children [59]. The positive parenting style of parents of children with chronic diseases may positively influence the parameters related to the child’s health. A previous study conducted with parents of children with chronic asthma reported that a positive parenting style was positively associated with the child’s general self-efficacy, medication compliance, and asthma control, whereas a negative parenting style was negatively associated [60]. A cross-sectional study conducted in China in 2019 evaluated the parenting style and various parental patterns of 236 parents with children diagnosed with chronic diseases and 98 parents with healthy children between the ages of 3 and 16. Parents of children with chronic illness had lower levels of the authoritative parenting style and family resilience than those of healthy children [61]. In a meta-analysis, the parent–child relationship, parenting behaviors, and styles of families with children diagnosed with chronic diseases were compared with those of families with healthy children [18]. Positive parent–child relationships tend to be lower in children with chronic illness, and lower parental responsiveness levels and higher demandingness and overprotective behavior levels have been observed in families with chronic disease. Moreover, higher authoritarian and neglectful parenting levels and lower authoritative parenting levels were observed in families with healthy children. This meta-analysis included studies with children with various chronic diseases and suggested that families of children with epilepsy, hearing impairment, and asthma struggle to identify appropriate levels of protective behavior, control, and parental warmth, as well as establish positive reciprocal relationships between parents and children [18]. In this study, although differences were noted between the groups regarding the relationship between the scores of the MAPS subscales and various variables of the parents of healthy children with epilepsy and healthy children, no difference was observed between parenting styles (*p* > 0.05), which may be because parents have similar sociocultural characteristics.

In the literature, no study has evaluated parenting style and mealtime behaviors together using the MAPS and PMAS as measurement tools. In 137 parent–child pairs evaluated using the Caregiver’s Feeding Styles Questionnaire and CEBQ, the mother’s demandingness during feeding was negatively associated with the child’s BMI for age Z scores and positively associated with slowness in eating and satiety responsiveness [62]. Maternal responsiveness was positively associated with the enjoyment of food and negatively associated with food fussiness. A positive relationship between the authoritarian behavior of mothers and food fussiness in children and a negative relationship between the authoritarian behavior of mothers and enjoyment of food behavior was shown [62]. A study was conducted in Brazil with parents of healthy children aged 1–7 years assessed using the Caregiver’s Feeding Styles Questionnaire and PMAS [28]. In the study, food refusal, food fussiness or pickiness of new and familiar foods, and neophobia were referred to as problematic eating behavior. Children of parents with indulgent parenting styles exhibited less problematic eating behaviors. Sharing the family menu with children and parents’ higher frequency of snacking restriction behavior were inversely associated with children’s problematic eating behavior [28]. In another study, 715 parents of children aged 3–5 years were administered the Caregivers’ Feeding Styles questionnaires. The results showed that parents’ indulgent styles were associated with a decrease in children’s intake of nutrient-rich foods [63]. In the present study, the PMAS snack model in the epilepsy group and the subdimension scores of reductions in animal fats in the control group were high. Furthermore, the snack modeling subscale score of PMAS in the epilepsy group and the fat reduction subscale score of the PMAS in the control group were high. This result is believed to be because the parents of the group with epilepsy had higher snacking habits owing to the emotional stress they experienced. It was suggested that the high educational level of the parents of the control group particularly reduced animal fat consumption by ensuring that their nutritional literacy was also high. In the control group, a positive correlation was noted between the broadband negative parenting subdimension score of the MAPS and the insistence on eating and snack modeling subscales of the PMAS and a negative correlation with the daily fruit–vegetable availability subscale of the PMAS. Despite the existence of controversial results in the literature, these results were consistent with those of other studies showing that negative parenting practices led to a decrease in healthy food consumption in healthy children.

In the literature, no study has examined the relationship between parenting style and eating behavior of children with epilepsy. However, in studies conducted with healthy children, emotional pressure to eat leads to negative effects in the direction of increased unhealthy food intake and decreased healthy food intake, whereas covert control and encouragement behaviors affect the child’s feeding behavior in the opposite direction [24,64,65]. A study evaluating 511 healthy preschool children aged 2–6 years using the CEBQ and Alabama Parenting Questionnaire reported that negative parenting styles were associated with children’s eating behavior [66]. Inconsistent parenting styles were positively associated with emotional overeating, food fussiness, and satiety responsiveness, whereas corporal punishment was positively associated with emotional overeating and food responsiveness but negatively associated with satiety responsiveness. No consistent relationship was noted between positive parenting styles and child eating behavior [66]. Using the Mealtime Assessment Survey and Parenting Styles and Dimensions Questionnaire, Podlesak et al. (2017) examined the relationship between parenting style and picky eating behaviors in healthy children aged 2–5 years and their parents [25]. Authoritarian and permissive parenting styles were positively associated with picky eating behaviors in children [25]. In this study, the fussiness subscale scores of the CEBQ did not differ between the groups. The scores of children with epilepsy who were reported to be picky eaters by their parents were higher than the scores of children who were not reported as picky eaters (*p* = 0.007 vs. *p* = 0.001, respectively), and the parents of these children had higher warmth subscale scores of the MAPS (*p* = 0.011). The higher prevalence of picky eating and satiety responsiveness behaviors including leaving food on the plate, not trying new foods, and getting full quickly in children with epilepsy may be because of the parents’ efforts to establish intimate relationships with their children owing to the disease burden. It is suggested that the positive relationship noted in the literature between negative parenting styles and picky eating behaviors is different between parents and children with chronic diseases. Despite the picky eating behavior in the epilepsy group, the HEI-2015 scores and BMI for age Z scores were similar to those of the healthy group. In this study, children with epilepsy had higher food approach behavior subdimension scores of the CEBQ, which is the sum of the food responsiveness, emotional overeating, enjoyment of food, and desire to drink subscales, than healthy children (*p* = 0.035). Moreover, in the epilepsy group, the broadband negative parenting subdimension of the MAPS increased food responsiveness, emotional overeating, and food approach behavior, and the broadband positive parenting subdimension increased satiety responsiveness behavior. In the control group, as broadband positive parenting increased, the emotional undereating behavior of children increased; as broadband negative parenting increased, the enjoyment of food behavior of children decreased, and the emotional overeating and food avoidant behavior of children increased. These results indicate that children with parents with negative parenting styles in the epilepsy group tended to have food approach behavior and higher food intake, which may be because of two reasons. Parents of children with epilepsy may overfeed their children to ensure healthy growth and development. This approach is supported by the fact that both groups had similar HEI-2015 scores and BMI for age Z scores. Nutrition is a biopsychological concept, and, besides being essential for growth and development in children, food consumption plays a hedonic role. Therefore, eating behaviors shaped by parents’ approach to their children may be related to the hedonic dimension of nutrition. In addition, in the regression analysis conducted to determine the independent factors affecting the level of food approach behavior in children with epilepsy, the subscales of the PMAS and broadband negative parenting explained 34% of the food approach behavior; broadband negative parenting and snack limits increased the food approach behavior level. These results suggest that negative parenting styles and parental attitudes toward snacking increase the child’s food intake; however, they also indicate that factors other than the scale variables play a role in the child’s eating behavior.

Complex relationships between epilepsy and the endocrine system exist, and growth retardation is expected in children with epilepsy [67]. Antiepileptic drugs used in epileptic seizure management have multisystemic effects, especially on the neurotransmission and endocrine systems. This interaction can alter the metabolism and absorption of several nutrients and the outcomes of eating behavior. Patients with epilepsy may, therefore, be at a higher risk of nutrient deficiency and its adverse effects [67,68]. Few studies on the relationship between antiepileptic drugs and appetite have suggested that serum ghrelin levels are increased in preadolescent children treated with valproic acid (VPA), which is frequently used in epilepsy treatment [67]. However, some studies have shown that ghrelin levels are reduced in adults with epilepsy and normal-weight preadolescent children receiving VPA treatment [69]. Berilgen et al. (2006) compared 35 patients receiving epilepsy treatment with a healthy control group in terms of ghrelin levels [70]. The epilepsy group had higher ghrelin levels. The origin of elevated serum ghrelin levels in epilepsy and their association with seizures are not fully understood [70]. In this study, children with epilepsy who received two or more antiepileptic drugs had lower slowness in eating subscale scores than those who received a single antiepileptic drug, and children who had seizures at least once a month had higher enjoyment of food subscale scores than those without seizures. It is suggested that the higher food approach behavior of children with epilepsy compared with healthy children is because of changes in ghrelin levels or medication use.

Children’s eating behaviors, which are shaped by parental attitudes and mealtime behaviors, affect the quality of their diet and, consequently, their growth. In a study of 99 parents in the United States, the parent–child relationship and child eating behaviors were assessed through food consumption records, the Caregiver’s Feeding Styles Questionnaire, BMI, and other demographic information [71]. Permissive feeding, wherein parents respond to their children’s requests while making few demands on them, was the most prevalent parental feeding style. This feeding approach has been linked to children’s consumption of low-nutrient-density foods [71]. In another study, the diet quality of children aged 9–15 years was assessed using the HEI-2005, and the Parenting Styles and Dimensions Questionnaire was administered to the parents [72]. As the dimensions of authoritative parenting style increased, children’s diet quality increased. Children of parents who exhibited permissive behaviors had lower HEI-2005 scores, and the freedom provided for children’s food choices may result in children selecting unhealthy foods [72]. In the present study, although the HEI-2015 scores did not differ between the groups, a positive correlation was noted between the positive reinforcement, supportiveness, and broadband positive parenting scores of the MAPS and HEI-2015 scores in the epilepsy group, which may be because of the relationship dynamics between the parent–child dyad with chronic disease unlike healthy children. Furthermore, snack limits and daily fruit–vegetable availability subscale scores of the PMAS and HEI-2015 in healthy children were positively correlated. However, another study suggested that parents limiting the amount of snacks and directing and pressuring children to consume fruits and vegetables can increase vegetable intake in children aged 5–11 years in the short term; however, in the long term, it can reduce the ability to regulate the amount of food eaten with internal satiety cues, increase eating when there is no hunger, and cause weight gain [73].

Studies have shown that authoritative parenting approaches, which include parental warmth and guidance, are associated with better body weight results than permissive or coercive parenting [24,65,74,75]. Another study reported a relationship between a positive feeding style and decreased satiety response and increased enjoyment of food, which are two aspects of self-regulation related to eating in children. Children of parents with indulgent feeding styles had higher body weights than children on other diets [76]. A study conducted with healthy children aged 6–10 years evaluated eating behaviors using the CEBQ and BMI for age Z scores. Overweight children had higher scores in the food-approach-related subdimensions and lower scores in the food-avoidance-related subdimensions [77]. Another study evaluated the eating behaviors of healthy children aged 6–12 years using the CEBQ [78]. Food responsiveness, which is associated with positive tendencies toward food consumption, and the enjoyment of food and emotional overeating subscale scores were strongly associated with childhood obesity, whereas negative relationships were noted between satiety responsiveness and the slowness in eating subscale scores and obesity [78]. In a study conducted on children with Tourette syndrome (TS), a neurological disorder, differences in food approach and food avoidant behaviors and their relationship with parental mealtime actions were assessed using the PMAS-Revised and CEBQ [42]. Children with TS exhibited higher food approach behavior, increased food responsiveness, emotional overeating, and a desire to drink compared to the control group. Although no significant difference in overall food avoidant behaviors was observed between the two groups, the TS group exhibited a considerably higher tendency for emotional overeating and fussiness. No significant difference in BMI was noted across the groups on the basis of age Z scores [42]. In a study conducted in Turkey, the CEBQ was administered to the parents of 520 healthy children aged 2–12 years, and the growth of the children was evaluated [79]. Although no difference was observed between normal and underweight children regarding CEBQ subscale scores, an inverse relationship was observed between the slowness in eating and the fussiness subscales in overweight children compared with the underweight group. An increase in the satiety responsiveness subscale scores increased the likelihood of a child being overweight by 1.3-fold [79]. A study by Coşkun et al. (2021), which evaluated the eating behavior in children with epilepsy and has the closest design to this study in the literature, compared the eating behavior differences in children with epilepsy and a healthy control group aged 6–16 years using the CEBQ [43]. No significant difference was noted between the CEBQ subscales for the two groups. However, the relationship between growth characteristics (BMI for age Z score) and CEBQ subscale scores was not evaluated [43]. In this study, no correlation was observed between BMI for age Z scores, the MAPS, and the PMAS subscale scores of children with epilepsy and healthy children; however, when children’s feeding behavior was evaluated, the food responsiveness and the enjoyment of food subscale scores of the CEBQ were positively correlated with BMI for age Z scores in children with epilepsy. However, the absence of growth differences noted between the epilepsy and healthy groups was evaluated as a positive result in this study, suggesting that parents with children with epilepsy attempted to feed their children in a way that prevents the negative effects of the disease.

This study had some limitations. First, although conducting the study in a single center allowed us to reach a sample with similar sociodemographic and cultural characteristics in terms of parenting style and eating behaviors, the sample size remained limited. Second, this study did not include details of pharmacologic treatments and biochemical parameters. The fact that antiepileptic drugs have different effects on the neuroendocrinologic system in the brain and that the patients were not evaluated in this respect was considered a limitation. Third, despite considerable evidence supporting the significance of parental feeding practices in the development of children’s eating behavior and obesity risk, observational studies are extremely limited, and data are obtained using validated questionnaires and scales. These instruments are based on parents’ and caregivers’ attitudes and statements about their own and their children’s eating behaviors. Lastly, as in all studies using this type of measurement tools, parents may not provide socially desirable answers about their own and their children’s behaviors and may not accurately remember how often they engage in certain behaviors.

## 5. Conclusions

To the best of our knowledge, this is the first study in the literature to examine the relationship between child eating behaviors, parenting style, and parental mealtime actions in epilepsy. No significant difference was noted between the parenting styles of families with healthy children and those with epilepsy. However, some differences were observed in both groups regarding the effects of parenting styles on children’s eating behaviors. The results of this study indicate that the reflection of the parent–child relationship on eating behaviors in children with chronic diseases is different from that in healthy children. However, no differences were noted in the diet quality and growth of the children. Studies aimed to develop strategies to provide an effective mealtime environment for parents and caregivers of children with chronic diseases and promote the development of healthy eating behaviors are warranted. Studies on this subject will have a significant role in improving the diet quality, ensuring the growth of the children, and improving the quality of life of the family. Parents’ negative parenting style increased food responsiveness, emotional overeating, and food approach behavior in children with epilepsy, whereas it negatively affected the enjoyment of food and increased food avoidant behavior in healthy children.

## Figures and Tables

**Table 1:** Demographic characteristics of parents.

	Epilepsy Group (*n* = 31)	Control Group (*n* = 31)	*p*-Value
	*n* (%)	*n* (%)	
Age (years), mean ± SD	37.71 ± 8.27	36.71 ± 7.19	0.532 ^a^
BMI (kg/m^2^) mean ± SD	26.60 ± 4.39	25.78 ± 5.79	0.613 ^a^
Sex			
Female	19 (61.3)	21(67.7)	0.596 ^b^
Male	12 (38.7)	10 (32.3)	
Marital status			
Married	29 (93.5)	27 (87.1)	0.671 ^c^
Single	2 (6.5)	4 (12.9)	
Educational level			
Primary	15 (48.4)	5 (16.1)	0.024 ^b,^*
High school	7 (22.6)	10 (32.3)	
University	9 (29)	16 (51.6)	
Occupational status			
Employed	13 (41.9)	16 (51.6)	0.445 ^b^
Unemployed	18 (58.1)	15 (48.4)	
Spousal consanguinity			
Yes	7 (22.6)	2 (6.5)	0.147 ^c^
No	24 (77.4)	29 (93.5)	
Income			
Less than expenses	3 (9.7)	3 (9.7)	0.435 ^c^
Equal to the expenses	25 (80.6)	21 (67.7)	
More than expenses	3 (9.7)	7 (22.6)	
Presence of the disease			
Yes	8 (25.8)	5 (16.1)	0.349 ^b^
No	23 (74.2)	26 (83.9)	

* *p* < 0.05. ^a^, independent-samples *t*-test; ^b^, Pearson’s chi-square test; ^c^, Fisher’s exact test. SD, standard deviation; BMI, body mass index.

**Table 2:** Demographic and medical characteristics of the children.

	Epilepsy Group (*n* = 31)	Control Group (*n* = 31)	*p*-Value
	*n* (%)	*n* (%)	
Age (years), mean ± SD	7.03 ± 1.33	6.48 ± 1.37	0.114
BMI Z score, mean ± SD	0.28 ± 1.40	−0.27 ± 1.61	0.158 ^a^
Age at diagnosis (months), mean ± SD	62.29 ± 32.98	N/A	N/A
Sex			
Female	16 (51.6)	14 (45.1)	0.258
Male	15 (48.4)	17 (54.9)	
Number of main meals			
2 meals	2 (6.5)	3 (9.6)	0.205 ^c^
3 meals	21 (67.7)	14 (45.2)	
>3 meals	8 (25.8)	14 (45.2)	
Picky eating status			
Yes	20 (64.5)	19 (61.3)	0.793 ^b^
No	11 (35.5)	12 (38.7)	
Number of antiepileptic drugs			
1	23 (74.2)	N/A	N/A
>1	8 (25.8)	N/A	
Seizures following pharmacologic treatment			
Yes	18 (58.1)	N/A	N/A
No	13 (41.9)	N/A	
Family history of epilepsy			
Yes	9 (29)	N/A	N/A
No	22 (71)	N/A	
Primary care provider			
Mother	19 (61.3)	N/A	N/A
Mother and father	9 (29)	N/A	
Other	3 (9.7)	N/A	

^a^, independent-samples *t*-test; ^b^, Pearson’s chi-square test; ^c^, Fisher’s exact test. SD, standard deviation; BMI, body mass index; N/A, not available.

**Table 3:** Scores of the scales and HEI-2015 according to the groups.

	Epilepsy Group (*n* = 31)	Control Group (*n* = 31)	*p*-Value ^a^
	Mean ± SD	Mean ± SD	
MAPS			
Proactive parenting	25.23 ± 2.85	26.00 ± 2.63	0.271
Positive reinforcement	17.74 ± 2.86	18.29 ± 1.90	0.378
Warmth	14.19 ± 1.28	14.39 ± 0.99	0.507
Supportiveness	36.39 ± 3.72	37.74 ± 3.15	0.127
Hostility	13.45 ± 3.80	14.39 ± 5.18	0.421
Lax control	14.65 ± 4.64	16.03 ± 5.98	0.312
Physical control	4.81 ± 1.64	5.13 ± 2.06	0.498
Broadband positive parenting	93.55 ± 8.12	96.42 ± 6.36	0.127
Broadband negative parenting	32.90 ± 7.38	35.55 ± 9.97	0.240
PMAS			
Snack limits	5.97 ± 1.92	6.55 ± 2.20	0.273
Positive persuasion	9.26 ± 2.03	9.42 ± 1.63	0.731
Daily fruit–vegetable availability	7.45 ± 1.34	7.52 ± 1.48	0.858
Use of rewards	7.03 ± 1.87	7.19 ± 1.68	0.722
Insistence on eating	5.06 ± 1.86	5.00 ± 1.63	0.885
Snack modeling	5.81 ± 1.72	4.97 ± 1.47	0.044 *
Special meals	8.94 ± 1.15	8.74 ± 1.00	0.483
Fat reduction	3.65 ± 1.14	4.48 ± 1.59	0.020 *
Many food choices	9.03 ± 1.76	8.65 ± 1.47	0.352
CEBQ			
Food responsiveness	9.77 ± 4.04	11.00 ± 4.27	0.250
Emotional overeating	6.65 ± 2.92	6.97 ± 2.63	0.649
Enjoyment of food	15.61 ± 5.35	15.39 ± 4.91	0.863
Desire to drink	9.84 ± 3.33	8.42 ± 3.97	0.132
Satiety responsiveness	21.81 ± 4.85	22.68 ± 5.83	0.525
Slowness in eating	9.90 ± 4.10	10.65 ± 4.55	0.503
Emotional undereating	10.58 ± 4.49	12.00 ± 4.20	0.204
Fussiness	8.16 ± 3.74	7.52 ± 3.23	0.471
Food approach behavior	10.31 ± 2.38	9.12 ± 1.98	0.035 *
Food avoidant behavior	10.96 ± 1.91	11.41 ± 2.45	0.424
HEI-2015	48.64 ± 11.51	46.19 ± 14.85	0.471

* *p* < 0.05. ^a^, independent-samples *t*-test. SD, standard deviation; MAPS, Multidimensional Assessment of Parenting Scale; PMAS, Parent Mealtime Action Scale; CEBQ, Children’s Eating Behavior Questionnaire; HEI-2015, Healthy Eating Index-2015.

**Table 4:** Evaluation of the relationship between MAPS, PMAS, and CEBQ scores.

	Epilepsy Group				Control Group			
	Broadband Positive Parenting		Broadband Negative Parenting		Broadband Positive Parenting		Broadband Negative Parenting	
	r	*p*-Value	r	*p*-Value	r	*p*-Value	r	*p*-Value
PMAS								
Snack limits	0.202	0.276	0.225	0.223	−0.164	0.377	−0.099	0.596
Positive persuasion	0.213	0.249	0.231	0.212	0.343	0.059	0.293	0.109
Daily fruit–vegetable availability	0.050	0.789	0.032	0.866	−0.300	0.101	−0.404	0.024 *
Use of rewards	0.049	0.792	0.073	0.698	0.251	0.173	0.218	0.238
Insistence on eating	−0.135	0.470	0.166	0.373	0.160	0.388	0.455	0.010 *
Snack modeling	−0.204	0.270	0.030	0.873	−0.027	0.885	0.435	0.014 *
Special meals	0.146	0.432	0.035	0.854	0.217	0.241	−0.270	0.142
Fat reduction	−0.011	0.955	0.043	0.817	−0.146	0.433	−0.171	0.358
Many food choices	−0.286	0.119	0.039	0.836	0.155	0.405	−0.034	0.856
CEBQ								
Food responsiveness	0.040	0.829	0.472	0.007 *	−0.031	0.870	−0.020	0.917
Emotional overeating	0.087	0.641	0.449	0.011 *	0.107	0.568	0.367	0.042 *
Enjoyment of food	−0.260	0.157	0.075	0.688	−0.208	0.261	−0.436	0.014 *
Desire to drink	0.186	0.317	0.246	0.181	0.260	0.158	0.085	0.649
Satiety responsiveness	0.431	0.015 *	−0.049	0.794	0.203	0.272	0.252	0.172
Slowness in eating	0.110	0.557	0.055	0.770	0.073	0.695	0.259	0.160
Emotional undereating	0.060	0.750	−0.022	0.905	0.372	0.040 *	0.445	0.012 *
Fussiness	−0.309	0.091	0.079	0.672	−0.025	0.892	−0.062	0.741
Food approach behavior	0.062	0.742	0.450	0.011 *	0.179	0.336	0.070	0.707
Food avoidant behavior	0.049	0.795	0.050	0.789	0.251	0.173	0.369	0.041 *

* *p* < 0.05. r, Pearson correlation test. PMAS, Parent Mealtime Action Scale; CEBQ, Children’s Eating Behavior Questionnaire.

**Table 5:** Evaluation of the relationships between HEI-2015 scores, BMI for age Z scores, and scale scores.

	HEI-2015 Score				BMI for Age Z Score			
	Epilepsy Group		Control Group		Epilepsy Group		Control Group	
	r	*p*-Value	r	*p*-Value	r	*p*-Value	r	*p*-Value
MAPS								
Proactive parenting	0.316	0.084	0.003	0.987	−0.040	0.831	0.242	0.189
Positive reinforcement	0.360	0.047 *	−0.140	0.453	0.006	0.972	−0.088	0.638
Warmth	0.274	0.135	−0.214	0.248	0.087	0.642	−0.028	0.883
Supportiveness	0.376	0.037 *	−0.268	0.144	−0.042	0.823	−0.152	0.415
Hostility	0.108	0.562	−0.065	0.730	−0.053	0.776	−0.232	0.209
Lax control	−0.068	0.716	−0.234	0.205	0.291	0.113	−0.141	0.450
Physical control	0.035	0.851	−0.170	0.360	−0.040	0.832	−0.140	0.453
Broadband positive parenting	0.453	0.011 *	−0.207	0.264	−0.017	0.927	−0.005	0.977
Broadband negative parenting	0.021	0.912	−0.209	0.259	0.147	0.431	−0.234	0.205
PMAS								
Snack limits	0.169	0.365	0.361	0.046 *	−0.007	0.970	−0.301	0.100
Positive persuasion	0.183	0.325	−0.164	0.378	−0.338	0.063	−0.210	0.256
Daily fruit–vegetable availability	0.233	0.207	0.427	0.017 *	−0.069	0.714	0.045	0.809
Use of rewards	0.083	0.659	0.132	0.480	−0.063	0.736	−0.314	0.085
Insistence on eating	0.020	0.917	0.164	0.379	−0.263	0.153	−0.172	0.355
Snack modeling	−0.013	0.944	−0.118	0.528	−0.177	0.341	−0.153	0.412
Special meals	0.114	0.542	−0.006	0.974	0.243	0.188	0.122	0.514
Fat reduction	0.234	0.206	0.037	0.845	0.318	0.081	−0.163	0.381
Many food choices	−0.051	0.786	−0.181	0.329	0.249	0.177	0.242	0.190
CEBQ								
Food responsiveness	0.054	0.772	0.242	0.189	0.431	0.016 *	−0.018	0.922
Emotional overeating	0.057	0.762	0.111	0.553	0.146	0.432	−0.024	0.899
Enjoyment of food	−0.011	0.953	0.267	0.146	0.470	0.008 *	0.342	0.059
Desire to drink	0.135	0.469	−0.223	0.227	−0.027	0.887	0.199	0.282
Satiety responsiveness	0.260	0.158	−0.339	0.062	−0.347	0.056	−0.018	0.923
Slowness in eating	0.101	0.590	−0.019	0.918	−0.344	0.058	−0.271	0.141
Emotional undereating	0.085	0.647	−0.144	0.441	−0.147	0.430	−0.136	0.467
Fussiness	−0.298	0.104	0.329	0.071	0.227	0.219	0.207	0.263
Food approach behavior	0.187	0.313	0.096	0.607	0.342	0.059	0.221	0.232
Food avoidant behavior	0.004	0.983	−0.041	0.827	−0.248	0.179	−0.099	0.597

* *p* < 0.05. r, Pearson correlation test. BMI, body mass index; HEI-2015, Healthy Eating Index-2015; MAPS, Multidimensional Assessment of Parenting Scale; PMAS, Parent Mealtime Action Scale; CEBQ, Children’s Eating Behavior Questionnaire.

**Table 6:** Evaluation of variables associated with the food approach behavior of the CEBQ.

			95% Confidence Interval						
Variables	B	SE	Lower	Upper	β	t	*p*	VIF	Tol.
Intercept	−9.55	5.12	−20.23	1.13		−1.866	0.077		
Broadband negative parenting	0.12	0.05	0.02	0.23	0.38	2.407	0.026 *	1.16	0.86
Snack limits	−0.03	0.21	−0.47	0.42	−0.02	−0.126	0.901	1.34	0.75
Positive persuasion	−0.05	0.21	−0.50	0.39	−0.04	−0.242	0.811	1.51	0.66
Daily fruit–vegetable availability	0.58	0.35	−0.14	1.31	0.33	1.670	0.111	1.74	0.58
Use of rewards	−0.48	0.26	−1.02	0.06	−0.38	−1.850	0.079	1.88	0.53
Insistence on eating	0.43	0.23	−0.05	0.91	0.34	1.865	0.077	1.48	0.67
Snack modeling	0.55	0.22	0.09	1.00	0.40	2.488	0.022 *	1.15	0.87
Special meals	0.72	0.40	−0.11	1.55	0.35	1.815	0.085	1.69	0.59
Fat reduction	0.71	0.35	−0.03	1.44	0.34	2.003	0.059	1.31	0.76
Many food choices	0.12	0.22	−0.34	0.58	0.09	0.554	0.586	1.19	0.84
Model summary	F(_10–20_)			2.55; *p* = 0.036					
	R^2^			0.560					
	Adjusted R^2^			0.341					
	DW statistic			2.11					
	Dependent variable			Food approach behavior					

* *p* < 0.05. B, estimates of unstandardized/standardized regression weights; β, estimates of standardized regression weights; SE, standard error; DW, Durbin–Watson statistic; VIF, variance inflation factor; Tol., tolerance value; CEBQ, Children’s Eating Behavior Questionnaire.

## Data Availability

The data supporting the findings of this study are available from the corresponding author, N.Ç.B., upon reasonable request. The data are not publicly available due to the participants have not given written consent for their data to be publicly shared.

## Supplementary Materials

The following supporting information can be downloaded at: https://www.mdpi.com/article/10.3390/nu16091384/s1, Table S1: The scales and HEI-2015 scores according to some characteristics of children with epilepsy; Table S2: The scales and HEI-2015 scores according to some characteristics of parents of children with epilepsy.
