# Supplementary material for: Evaluation of the Effect of Parenting Style and Parental Mealtime Actions on the Eating Behavior of Children with Epilepsy
Source: Nutrients. 2024 May 2;16(9):1384. doi: 10.3390/nu16091384 (PMC11085228; doi:10.3390/nu16091384)
Supplement: Supplementary file 1 [file nutrients-16-01384-s001.zip › nutrients-2913309-supplementary.pdf]

Evaluation of the Effect of Parenting Style and Parental Eating Time Actions on Eating Behavior in Children with Epilepsy

Table S1: The scales and HEI-2015 scores according to some characteristics of children with epilepsy.

	**Age**			**Number of antiepileptic drugs**			**Seizures after pharmacologic treatment**			**Picky eating status**		
	**≤5.4 years**	**>5.4 years**		**I**	**≥II**		**Yes**	**No**		**Yes**	**No**	
	**Mean ± SD**	**Mean ± SD**	**p-value**	**Mean ± SD**	**Mean ± SD**	**p-value**	**Mean ± SD**	**Mean ± SD**	**p-value**	**Mean ± SD**	**Mean ± SD**	**p-value**
MAPS												
Proactive parenting	25.07 ± 3.25	25.35 ± 2.57	0.789	24.96 ± 2.85	26.00 ± 2.88	0.381	24.61 ± 2.81	26.08 ± 2.78	0.161	25.55 ± 3.09	24.64 ± 2.38	0.402
Positive reinforcement	18.21 ± 2.15	17.35 ± 3.35	0.414	17.48 ± 3.16	18.50 ± 1.69	0.394	18.00 ± 1.85	17.39 ± 3.93	0.564	18.00 ± 3.21	17.27 ± 2.15	0.508
Warmth	14.00 ± 1.36	14.35 ± 1.22	0.453	14.09 ± 1.35	14.50 ± 1.07	0.440	13.89 ± 1.53	14.62 ± 0.65	0.119	14.75 ± 0.44	13.18 ± 1.66	0.011*
Supportiveness	37.50 ± 4.03	35.47 ± 3.28	0.133	35.61 ± 3.50	38.63 ± 3.62	0.046*	36.72 ± 3.36	35.92 ± 4.27	0.564	36.40 ± 3.78	36.36 ± 3.80	0.980
Hostility	12.57 ± 2.79	14.18 ± 4.42	0.249	13.61 ± 3.99	13.00 ± 3.42	0.703	12.56 ± 2.75	14.69 ± 4.75	0.124	13.65 ± 3.50	13.09 ± 4.46	0.702
Lax control	13.71 ± 4.20	15.41 ± 4.98	0.319	14.83 ± 5.19	14.13 ± 2.70	0.720	14.67 ± 4.64	14.62 ± 4.84	0.976	15.10 ± 4.12	13.82 ± 5.60	0.472
Physical control	5.29 ± 2.02	4.41 ± 1.18	0.143	4.48 ± 1.16	5.75 ± 2.43	0.192	4.89 ± 1.84	4.69 ± 1.38	0.748	4.40 ± 0.99	5.55 ± 2.30	0.141
Broadband positive parenting	94.79 ± 8.71	92.53 ± 7.72	0.451	92.13 ± 8.44	97.63 ± 5.78	0.100	93.22 ± 7.07	94.00 ± 9.69	0.798	94.70 ± 8.36	91.45 ± 7.61	0.295
Broadband negative parenting	31.57 ± 5.29	34.00 ± 8.75	0.371	32.91 ± 8.22	32.88 ± 4.58	0.990	32.11 ± 5.08	34.00 ± 9.87	0.491	33.15 ± 5.07	32.45 ± 10.68	0.807
PMAS												
Snack limits	5.36 ± 1.87	6.47 ± 1.88	0.110	6.00 ± 1.95	5.88 ± 1.96	0.877	6.11 ± 1.91	5.77 ± 2.01	0.633	5.65 ± 1.69	6.55 ± 2.25	0.221
Positive persuasion	9.79 ± 1.72	8.82 ± 2.22	0.194	9.22 ± 2.26	9.38 ± 1.30	0.854	9.22 ± 2.05	9.31 ± 2.10	0.910	9.50 ± 2.12	8.82 ± 1.89	0.380
Daily fruit-vegetable availability	7.07 ± 1.64	7.76 ± 0.97	0.178	7.35 ± 1.43	7.75 ± 1.04	0.473	7.11 ± 1.57	7.92 ± 0.76	0.067	7.70 ± 1.22	7.00 ± 1.48	0.167
Use of rewards	7.00 ± 1.62	7.06 ± 2.11	0.932	6.91 ± 2.04	7.38 ± 1.30	0.556	6.78 ± 1.99	7.38 ± 1.71	0.382	7.40 ± 1.85	6.36 ± 1.80	0.143
Insistence on eating	4.57 ± 1.22	5.47 ± 2.21	0.164	5.26 ± 2.03	4.50 ± 1.20	0.327	4.78 ± 1.80	5.46 ± 1.94	0.321	5.05 ± 2.01	5.09 ± 1.64	0.954
Snack modelling	6.14 ± 1.56	5.53 ± 1.84	0.332	6.00 ± 1.78	5.25 ± 1.49	0.296	5.89 ± 1.64	5.69 ± 1.89	0.759	5.80 ± 1.82	5.82 ± 1.60	0.978
Special meals	9.29 ± 1.07	8.65 ± 1.17	0.127	8.87 ± 1.18	9.13 ± 1.13	0.598	9.06 ± 1.26	8.77 ± 1.01	0.504	9.15 ± 0.88	8.55 ± 1.51	0.242
Fat reduction	3.50 ± 1.02	3.76 ± 1.25	0.530	3.61 ± 1.03	3.75 ± 1.49	0.769	3.83 ± 1.25	3.38 ± 0.96	0.288	3.50 ± 0.95	3.91 ± 1.45	0.348
Many food choices	9.00 ± 2.00	9.06 ± 1.60	0.928	9.13 ± 1.79	8.75 ± 1.75	0.607	9.33 ± 1.28	8.62 ± 2.26	0.316	9.05 ± 2.06	9.00 ± 1.10	0.930
CEBQ												
Food responsiveness	9.29 ± 3.56	10.18 ± 4.46	0.550	9.78 ± 4.42	9.75 ± 2.92	0.985	10.44 ± 3.68	8.85 ± 4.47	0.284	9.25 ± 3.42	10.73 ± 5.02	0.338
Emotional overeating	6.50 ± 3.06	6.76 ± 2.88	0.806	6.87 ± 3.27	6.00 ± 1.51	0.477	6.61 ± 2.87	6.69 ± 3.09	0.941	5.90 ± 1.83	8.00 ± 4.00	0.124
Enjoyment of food	15.21 ± 4.74	15.94 ± 5.93	0.713	15.17 ± 5.98	16.88 ± 2.80	0.448	17.39 ± 5.08	13.15 ± 4.88	0.027*	14.10 ± 5.58	18.36 ± 3.72	0.031*
Desire to drink	9.57 ± 3.55	10.06 ± 3.23	0.692	9.74 ± 3.51	10.13 ± 2.95	0.783	9.83 ± 3.57	9.85 ± 3.11	0.992	10.25 ± 3.55	9.09 ± 2.88	0.362
Satiety responsiveness	22.07 ± 4.27	21.59 ± 5.41	0.788	22.30 ± 5.17	20.38 ± 3.70	0.341	20.78 ± 3.98	23.23 ± 5.72	0.169	23.80 ± 4.26	18.18 ± 3.71	0.001*

Slowness in eating	10.64 ± 4.40	9.29 ± 3.87	0.371	10.83 ± 3.99	7.25 ± 3.37	0.031*	9.33 ± 4.06	10.69 ± 4.19	0.372	10.35 ± 4.67	9.09 ± 2.81	0.357
Emotional undereating	11.07 ± 4.81	10.18 ± 4.31	0.589	10.61 ± 4.64	10.50 ± 4.31	0.954	10.72 ± 4.61	10.38 ± 4.48	0.840	10.65 ± 4.34	10.45 ± 4.95	0.910
Fussiness	8.21 ± 3.31	8.12 ± 4.17	0.944	8.17 ± 3.89	8.13 ± 3.52	0.975	9.11 ± 3.20	6.85 ± 4.16	0.097	10.27 ± 2.33	7.00 ± 3.91	0.007*
Food approach behavior	10.08 ± 2.39	10.50 ± 2.42	0.633	10.35 ± 2.58	10.20 ± 1.82	0.879	10.72 ± 2.49	9.75 ± 2.19	0.270	10.02 ± 2.06	10.85 ± 2.90	0.362
Food avoidant behavior	11.32 ± 1.84	10.66 ± 1.98	0.346	11.27 ± 1.94	10.06 ± 1.63	0.124	11.02 ± 2.06	10.87 ± 1.76	0.835	10.98 ± 1.99	10.91 ± 1.85	0.919
HEI-2015	50.29 ± 13.70	47.28 ± 9.58	0.479	47.52 ± 10.12	51.87 ± 15.17	0.366	46.33 ± 10.25	51.84 ± 12.79	0.193	50.29 ± 12.31	45.63 ± 9.72	0.288

*p<0.05, a: Independent samples t test

SD: Standard deviation; MAPS: The Multidimensional Assessment of Parenting Scale; PMAS: The Parent Mealtime Action Scale; CEBQ: The Children's Eating Behavior Questionnaire; HEI-2015: The Healthy Eating Index-2015

Table S2: The scales and HEI-2015 scores according to some characteristics of parents of children with epilepsy.

	Age			Sex			Education level		
	< 40 years	≥ 40 years		Female	Male		Primary school	≥ High school	
	**Mean ± SD**	**Mean ± SD**	**p-value**	**Mean ± SD**	**Mean ± SD**	**p-value**	**Mean ± SD**	**Mean ± SD**	**p-value**
MAPS									
Proactive parenting	25.33 ± 3.25	25.08 ± 2.29	0.809	25.25 ± 2.45	25.21 ± 3.14	0.971	24.33 ± 2.94	26.06 ± 2.57	0.091
Positive reinforcement	17.50 ± 3.33	18.08 ± 2.14	0.588	17.00 ± 4.05	18.21 ± 1.75	0.258	17.60 ± 2.10	17.88 ± 3.50	0.794
Warmth	14.17 ± 1.47	14.23 ± 1.01	0.893	14.33 ± 0.89	14.11 ± 1.49	0.636	13.67 ± 1.63	14.69 ± 0.48	0.033*
Supportiveness	35.83 ± 3.73	37.15 ± 3.72	0.338	35.25 ± 4.25	37.11 ± 3.26	0.181	36.27 ± 3.94	36.50 ± 3.63	0.865
Hostility	14.28 ± 4.36	12.31 ± 2.59	0.158	12.50 ± 3.73	14.05 ± 3.82	0.275	12.20 ± 4.11	14.63 ± 3.18	0.075
Lax control	15.56 ± 5.51	13.38 ± 2.82	0.164	13.75 ± 3.98	15.21 ± 5.04	0.403	16.07 ± 5.54	13.31 ± 3.26	0.100
Physical control	4.72 ± 1.32	4.92 ± 2.06	0.743	4.33 ± 0.89	5.11 ± 1.94	0.144	5.33 ± 2.09	4.31 ± 0.87	0.096
Broadband positive parenting	92.83 ± 8.96	94.54 ± 7.03	0.573	91.83 ± 9.75	94.63 ± 6.98	0.359	91.87 ± 8.40	95.13 ± 7.79	0.272
Broadband negative parenting	34.56 ± 8.39	30.62 ± 5.16	0.145	30.58 ± 6.30	34.37 ± 7.78	0.168	33.60 ± 9.17	32.25 ± 5.42	0.619
PMAS									
Snack limits	6.17 ± 1.98	5.69 ± 1.89	0.507	5.67 ± 2.15	6.16 ± 1.80	0.498	6.40 ± 1.96	5.56 ± 1.86	0.232
Positive persuasion	9.39 ± 2.25	9.08 ± 1.75	0.681	8.58 ± 2.54	9.68 ± 1.57	0.145	8.93 ± 1.87	9.56 ± 2.19	0.398
Daily fruit-vegetable availability	7.44 ± 1.38	7.46 ± 1.33	0.973	7.08 ± 1.68	7.68 ± 1.06	0.283	7.27 ± 1.58	7.63 ± 1.09	0.465
Use of rewards	7.06 ± 1.89	7.00 ± 1.92	0.937	6.50 ± 1.88	7.37 ± 1.83	0.213	6.93 ± 2.28	7.13 ± 1.46	0.781
Insistence on eating	4.94 ± 1.70	5.23 ± 2.13	0.680	4.75 ± 1.22	5.26 ± 2.18	0.408	5.13 ± 1.51	5.00 ± 2.19	0.846
Snack modelling	6.39 ± 1.75	5.00 ± 1.35	0.024*	5.50 ± 1.98	6.00 ± 1.56	0.440	5.93 ± 1.71	5.69 ± 1.78	0.698
Special meals	8.83 ± 1.25	9.08 ± 1.04	0.570	9.17 ± 1.47	8.79 ± 0.92	0.384	9.00 ± 1.36	8.88 ± 0.96	0.769
Fat reduction	3.61 ± 1.09	3.69 ± 1.25	0.849	3.83 ± 1.27	3.53 ± 1.07	0.475	3.80 ± 1.08	3.50 ± 1.21	0.474
Many food choices	9.11 ± 1.57	8.92 ± 2.06	0.775	9.42 ± 1.56	8.79 ± 1.87	0.342	9.27 ± 1.03	8.81 ± 2.26	0.475
CEBQ									
Food responsiveness	9.89 ± 4.86	9.62 ± 2.69	0.856	7.75 ± 2.73	11.05 ± 4.26	0.024*	10.60 ± 4.44	9.00 ± 3.60	0.278
Emotional overeating	6.89 ± 3.58	6.31 ± 1.70	0.593	6.17 ± 3.07	6.95 ± 2.86	0.477	7.40 ± 3.80	5.94 ± 1.57	0.182
Enjoyment of food	15.67 ± 4.85	15.54 ± 6.19	0.949	15.00 ± 5.74	16.00 ± 5.22	0.621	17.60 ± 4.64	13.75 ± 5.43	0.043*
Desire to drink	10.78 ± 3.10	8.54 ± 3.31	0.063	9.00 ± 3.72	10.37 ± 3.04	0.272	10.27 ± 3.22	9.44 ± 3.48	0.497
Satiety responsiveness	22.33 ± 4.64	21.08 ± 5.24	0.486	21.75 ± 5.48	21.84 ± 4.57	0.960	19.80 ± 3.80	23.69 ± 5.08	0.023*
Slowness in eating	10.39 ± 3.99	9.23 ± 4.32	0.447	9.83 ± 4.67	9.95 ± 3.84	0.941	8.40 ± 3.60	11.31 ± 4.14	0.046*
Emotional undereating	9.67 ± 3.85	11.85 ± 5.13	0.186	8.83 ± 4.39	11.68 ± 4.30	0.085	10.93 ± 4.77	10.25 ± 4.33	0.679
Fussiness	8.06 ± 3.40	8.31 ± 4.31	0.857	7.75 ± 3.89	8.42 ± 3.73	0.635	6.69 ± 3.93	9.73 ± 2.89	0.020*
Food approach behavior	10.43 ± 2.90	10.16 ± 1.47	0.739	9.52 ± 2.26	10.81 ± 2.37	0.143	10.91 ± 2.73	9.75 ± 1.91	0.179

Food avoidant behavior	10.89 ± 1.80	11.05 ± 2.13	0.823	10.36 ± 1.60	11.34 ± 2.04	0.170	10.91 ± 1.87	11.00 ± 2.01	0.890
HEI-2015	46.40 ± 10.75	51.75 ± 12.23	0.207	48.34 ± 11.62	48.83 ± 11.76	0.911	47.56 ± 11.91	49.65 ± 11.42	0.622

p<0.05, a: Independent samples t test

SD: Standard deviation; MAPS: The Multidimensional Assessment of Parenting Scale; PMAS: The Parent Mealtime Action Scale; CEBQ: The Children's Eating Behavior Questionnaire; HEI-2015: The Healthy Eating Index-2015
